# Hardtank^®^ technology in extraction of caffeine and chlorogenic acid from coffee: analysis of chemical composition and antioxidant properties of coffee beverages

**DOI:** 10.3389/fchem.2025.1681137

**Published:** 2025-09-26

**Authors:** Radosław Kowalski, Kamil Belniak, Grażyna Kowalska, Marzena Włodarczyk-Stasiak, Artur Mazurek, Tomasz Baj

**Affiliations:** 1 Department of Analysis and Evaluation of Food Quality, University of Life Sciences in Lublin, Lublin, Poland; 2 Department of Tourism and Recreation, University of Life Sciences in Lublin, Lublin, Poland; 3 Department of Pharmacognosy with Medicinal Plants Garden, Medical University of Lublin, Lublin, Poland

**Keywords:** antioxidant activity, caffeine, chlorogenic acid, cold brew coffee, hardtank^®^, phenolic compounds

## Abstract

**Introduction:**

Hardtank® is a pressure-modulated, solvent-free cold extraction that shortens brewing while maximizing bioactive recovery from roasted coffee.

**Methods:**

We produced a Nitro Cold Brew beverage from roasted Yellow Bourbon beans using Hardtank® (550 g : 10,000 mL water, 30 min, 0.3–1.5 bar) and compared its composition and antioxidant activity with laboratory extracts from roasted and green beans obtained by hot water infusion (93 °C, 5 min) and by methanol extraction (70% reflux, 2 h; 100% reflux, 2 h; 70% shake, 5 min). We measured caffeine, chlorogenic acid (and derivatives), total phenolics, total flavonoids, and antioxidant activity by the ferric reducing antioxidant power and DPPH radical tests, expressed as Trolox equivalents.

**Results and discussion:**

Nitro Cold Brew contained 375.8±2.4 mg phenolics + flavonoids per 100 mL, 72.4±1.1 mg caffeine per 100 mL, and 78.2±2.3 mg chlorogenic acid per 100 mL, with ferric reducing antioxidant power 27.9±0.5 mM and DPPH 11.7±0.6 mM. Using 70% methanol reflux as the 100% reference, Hardtank® achieved 135.3% for phenolics + flavonoids, 119.7% for caffeine, and up to 172.3% for chlorogenic acid. Overall, Hardtank® yielded a ready-to-drink coffee with bioactive and antioxidant profiles comparable to or better than laboratory reflux extracts, while operating rapidly and without organic solvents, indicating promise for scalable production of functional coffee beverages.

## 1 Introduction

Coffee beans became widely recognized as an industrial product in the second half of the 19th century, when rapid growth in production in Brazil contributed to the development of a mass consumer market in the United States. Coffee is one of the most popular beverages worldwide, highly valued and consumed in diverse settings, such as during meals, work breaks, and social gatherings (Gotteland and de Pablo, 2007). It is characterized not only by attractive sensory features but also by stimulating, nutritional, and health-promoting properties, resulting from the presence of bioactive compounds such as caffeine, polyphenols, diterpenes, and melanoids (de Oliveira et al., 2021; Morris, 2019; Silva et al., 2020; Wu et al., 2022). Studies conducted by Zhang et al. (2021) on a group of over 365,000 participants found that regular consumption of coffee infusions may reduce the risk of post-stroke dementia due to the presence of bioactive ingredients. The content of these active substances in coffee largely depends on the extraction method used. Coffee can be prepared using various techniques, ranging from simple brewing methods requiring no special equipment to advanced processes employed by professionals in coffee shops or industrial facilities using specialized apparatus. Depending on the brewing method, the chemical composition of the coffee infusion can vary significantly, influencing both its sensory quality and health benefits.

Caffeine (CAF), the most commonly consumed psychoactive substance worldwide, has been associated with numerous health benefits, including a reduced risk of Parkinson’s disease and type 2 diabetes. While coffee infusions are the primary source of CAF globally, exceptions exist in regions with a strong tea-drinking culture (Chen, 2022). In the United States, energy drinks were introduced in 1997 as stimulants or dietary supplements, with CAF content ranging from 50 mg to 505 mg, compared to the typical 30–80 mg in standard coffee beverages (Olas and Bryś, 2019). CAF has been shown to improve mood, well-being, athletic performance, information processing speed, awareness, attention, and reaction time. Research suggests it may also alleviate Parkinson’s disease symptoms, such as impaired motor skills and tremors (Heckman et al., 2010). Moreover, consuming caffeinated beverages like coffee and tea may reduce the risk of cardiovascular disease (CVD) by exerting anti-inflammatory, antioxidant, and blood sugar-lowering effects. CVD remains one of the most prevalent chronic diseases globally and the leading cause of premature death and disability (Zheng et al., 2022). However, CAF consumption during pregnancy may increase the risk of very low birth weight infants (Liao et al., 2022). Studies also highlight an inverse relationship between coffee consumption and health issues such as diabetes, cancer, Alzheimer’s, and Parkinson’s disease, linked to the activity of phenolic compounds (Pimpley and Murthy, 2021). Although moderate coffee consumption is associated with health benefits, excessive consumption may carry a risk for the cardiovascular system. Studies have shown that the diterpenes found in coffee–cafestol and kahweol–increase total and LDL cholesterol levels, which translates into an increased risk of atherosclerosis (Urgert et al., 1995). Furthermore, long-term and high CAF consumption may lead to a transient increase in blood pressure, which increases the risk of cardiovascular events in people with hypertension (Ranheim and Halvorsen, 2005).

Chlorogenic acid (ChA) is one of the principal phenolic compounds in coffee, comprising 4%–8.4% of the dry weight of Arabica beans and up to 14.4% in Robusta (Farah and Donangelo, 2006). Its structure–as an ester of caffeic and quinic acids–confers strong antioxidant properties, allowing ChA to neutralize free radicals and protect lipids and proteins from oxidation. In coffee brews, ChA content depends on bean variety, roast level, and brewing parameters; in espresso (7 g/30 mL, 9 bar) ChA typically ranges from 30 to 140 mg per cup, whereas cold brew prepared over 12–24 h can deliver 150–300 mg ChA per cup, depending on maceration time and roast degree (Moon and Shibamoto, 2009; Rao et al., 2020). *In vitro* studies indicate that ChA exhibits anti-inflammatory and hypoglycemic effects, including inhibition of α-glucosidase activity and reduction of oxidative stress in endothelial cells (Olthof et al., 2001; Tajik et al., 2017). Moreover, clinical data suggest that regular consumption of ChA -rich coffee may reduce cardiovascular disease risk and improve glycemic profiles in individuals predisposed to insulin resistance (Malerba et al., 2013; O’Keefe et al., 2013; Watanabe et al., 2021). Due to these unique properties, ChA serves not only as a marker of coffee quality but also as a promising target for developing functional beverages and extracts with health-promoting effects (Corso et al., 2016). In the present study, we examine the impact of various extraction techniques–including the novel Hardtank^®^ technology–on ChA yield and its antioxidant potential in the final brew.

The brewing of coffee beans is essentially a solid-liquid extraction process, where water-soluble compounds from ground coffee beans (roasted or green) are eluted into the infusion. To enhance extraction efficiency, roasted coffee beans are ground and exposed to hot water. The extraction time ranges from 20 s to several minutes, depending on particle size and the brewing technique (Zhang et al., 2022b).

Recently, the food industry has witnessed a surge in the popularity of cold brew coffee, including iced coffee with ice cubes and cold brew drinks obtained through cold extraction. Iced coffee now accounts for over 20% of new products. Consumer preference studies have identified cost and calorific value as the most critical factors influencing purchase decisions. Understanding consumer behavior and preferences for iced coffee is key to market success in the coming years (Beekman et al., 2021).

Over the past decade, cold brew coffee has gained widespread consumer acceptance. This method produces coffee with a sweeter, less bitter taste compared to traditional brewing methods. Unlike conventional techniques, cold brew involves steeping ground coffee beans in water at cold to room temperatures for 6–24 h. The extraction time significantly affects the sensory qualities of the beverage, including CAF and ChA content. The equilibrium of these compounds is typically achieved between 6 and 7 h of water maceration at 21 °C–25 °C. Both maceration temperature (4 °C–93 °C) and particle size (139–1747 μm) influence extraction kinetics. Reducing particle size can substantially enhance extraction efficiency, similar to increasing the temperature of the extractant. Additionally, techniques like ultrasonication, stirring, and shaking can further improve the extraction process (Zhang et al., 2022b).

The innovative Hardtank^®^ (HT) (percolated cold brew) device offers an alternative to traditional 24-h cold brew maceration. The HT device utilizes a carefully designed hydrodynamic system to perform maceration and percolation of ground coffee beans, reducing extraction time to 30–50 min while achieving optimal efficiency. Particle size plays a crucial role in this process, affecting the formation of the coffee particle bed in the flotation basket (Stanek et al., 2021). Coffee beverages produced with HT technology are already commercially available. Consumers often select products based on advertising, trends, and prevailing market fads. However, the labels of such products typically provide only nutritional and CAF content, omitting information about other biologically active compounds that contribute to their health-promoting properties. Therefore, studies characterizing the biologically active compounds in these new beverages, compared to those in traditional coffee infusions, are of significant interest.

In light of this, the present study evaluated the composition of a commercial cold brew coffee beverage produced using HT technology, comparing it with classic coffee infusions. The analysis included total phenolic compounds (TPC), total flavonoids (TFC), CAF, ChA, CA and derivatives of these phenolic acids (ChAd and CAd).

## 2 Materials and methods

### 2.1 Chemicals

All chemicals used in this study were of analytical grade or higher. Methanol (HPLC grade, ≥99.9%) and methanol (analytical grade, 99.8%) were used for extractions and chromatographic analyses. Ethanol (HPLC grade, ≥99.9%), orthophosphoric acid (ACS reagent, ≥85%), hydrochloric acid (ACS reagent, 37%), Folin–Ciocalteu reagent, sodium carbonate (≥99.5%, ACS), sodium nitrate (≥99.0%, ACS), aluminum chloride hexahydrate (≥98.0%, ACS), acetate buffer components, 2,4,6-tripyridyl-s-triazine (TPTZ, ≥98%), DPPH (2,2-diphenyl-1-picrylhydrazyl, ≥95%), iron (III) chloride hexahydrate (FeCl_3_·6H_2_O, ≥99%), and iron (II) sulfate heptahydrate (FeSO_4_·7H_2_O, ≥99%) were purchased from Sigma-Aldrich (St. Louis, MO, United States), unless otherwise specified.

Reference standards for HPLC analysis, including caffeine (CAF, ≥99%, HPLC grade), chlorogenic acid (ChA, ≥95%, HPLC grade), caffeic acid (CA, ≥98%, HPLC grade), p-coumaric acid (pCA, ≥98%, HPLC grade), and Trolox (≥97%, HPLC grade), as well as calibration standards for atomic absorption spectroscopy (AAS), were also obtained from Sigma-Aldrich. Gallic acid (GAE, ≥98%, ACS) and epicatechin (ECE, ≥98%, HPLC grade) were used as calibration standards for TPC and TFC, respectively.

All aqueous solutions were prepared using high-purity deionized water (resistivity 18.2 MΩ cm, deionization system HLP 5, Hydrolab, Straszyn, Poland), produced by reverse osmosis followed by ion-exchange cartridges.

### 2.2 Coffee beans

The research material consisted of green coffee beans provided by Hard Beans Sp. z o.o. (Opole, Poland). The beans originated from Brazil, specifically the Campo das Vertentes region, and were produced by Henrique Dias Cambraia. The harvest took place in 2022 and in 2024 at an altitude of 900 m above sea level. The beans were processed using the pulped natural method (15–20 days on raised beds) and belonged to the Yellow Bourbon variety. Subsequently, the coffee beans were roasted at Hard Beans Sp. z o.o. (Opole, Poland) on November 30, 2022 (batch 1, harvest July 2022) and on February 4, 2025 (batch 2, harvest July 2024).

### 2.3 Coffee drink

**FIGURE 1:**
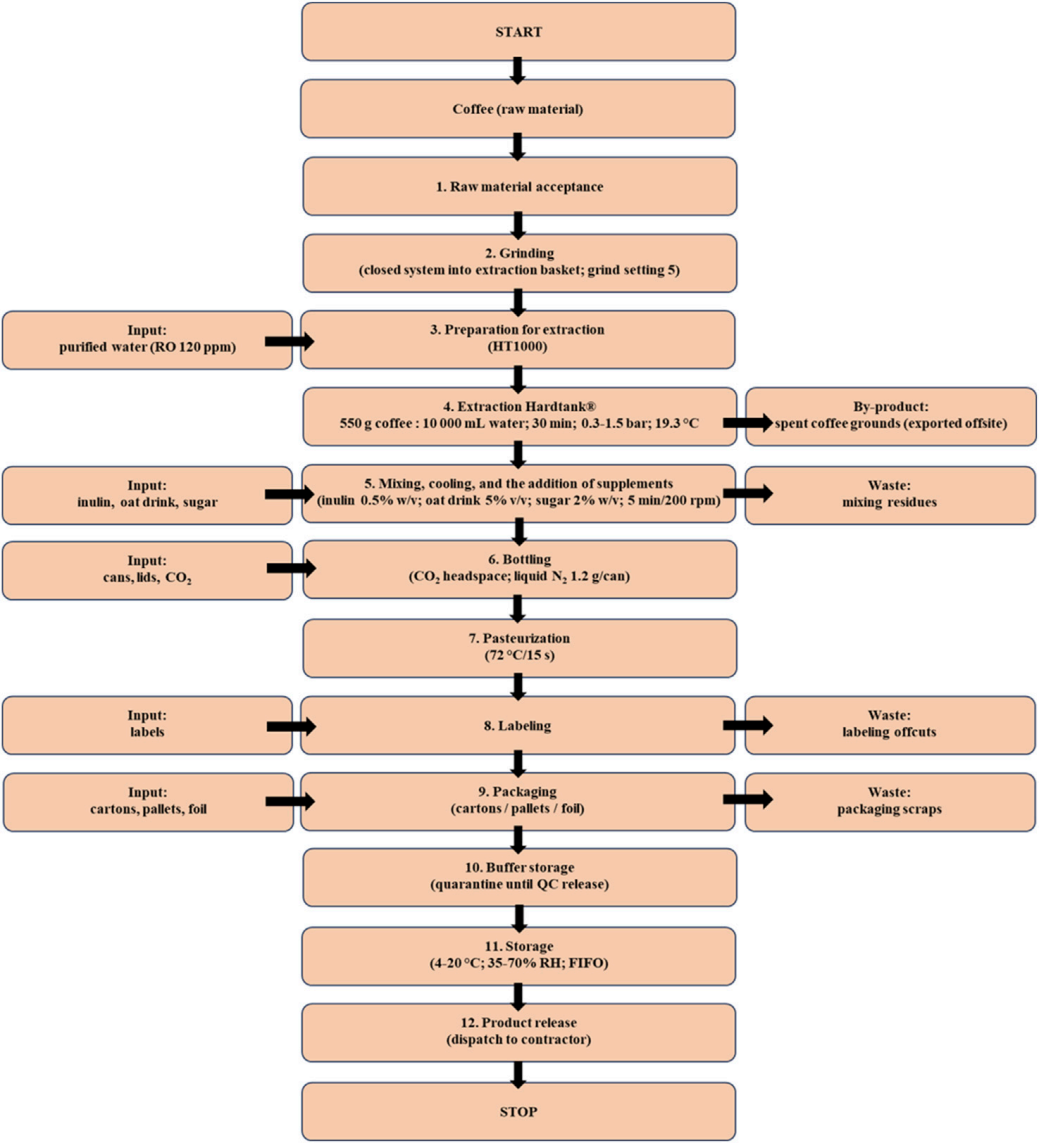
Technological diagram of coffee beverage production using Hardtank^®^ technology.

The roasted coffee beans were subjected to extraction using the Hardtank^®^ (HT) method (HT1000, Hard Beans sp. z o.o., Opole, Poland), resulting in the commercial Nitro Cold Brew coffee drink produced by Hard Beans Sp. z o.o. (Opole, Poland). Coffee beans, described in chapter 2.2, were used to produce the coffee beverage. The production process of the beverage is illustrated in Figure 1.

HT coffee drinks were prepared using 550.0 g of ground coffee (Mahlkoenig VTA 6S, grind setting: 5) and 10,000 mL of RO AKVO 120 ppm quality water at a temperature of 19.3 °C (Fetco, 2024; Hard Beans, 2023; Hard Beans, 2025). RO AKVO 120 ppm quality water refers to reverse osmosis (RO) purified water standardized to 120 ppm total dissolved solids (TDS), with low mineral content and stable physicochemical parameters, identical to that used by the manufacturer in commercial production. The maceration and percolation process was carried out over 30 min at a modulated pressure of 0.3–1.5 bar (Stanek et al., 2021).

Nitro Cold Brew is a creamy coffee beverage produced in two stages.

1. Cold water extraction in the patented Hardtank^®^ system (Matejuk et al., 2020), which, through dynamic circulation and pressure modulation, yields an extract rich in bioactive compounds in 30–50 min.
2. Saturation of the finished extract with liquid nitrogen during bottling, which stabilizes the product and gives it a creamy texture.

**FIGURE 2:**
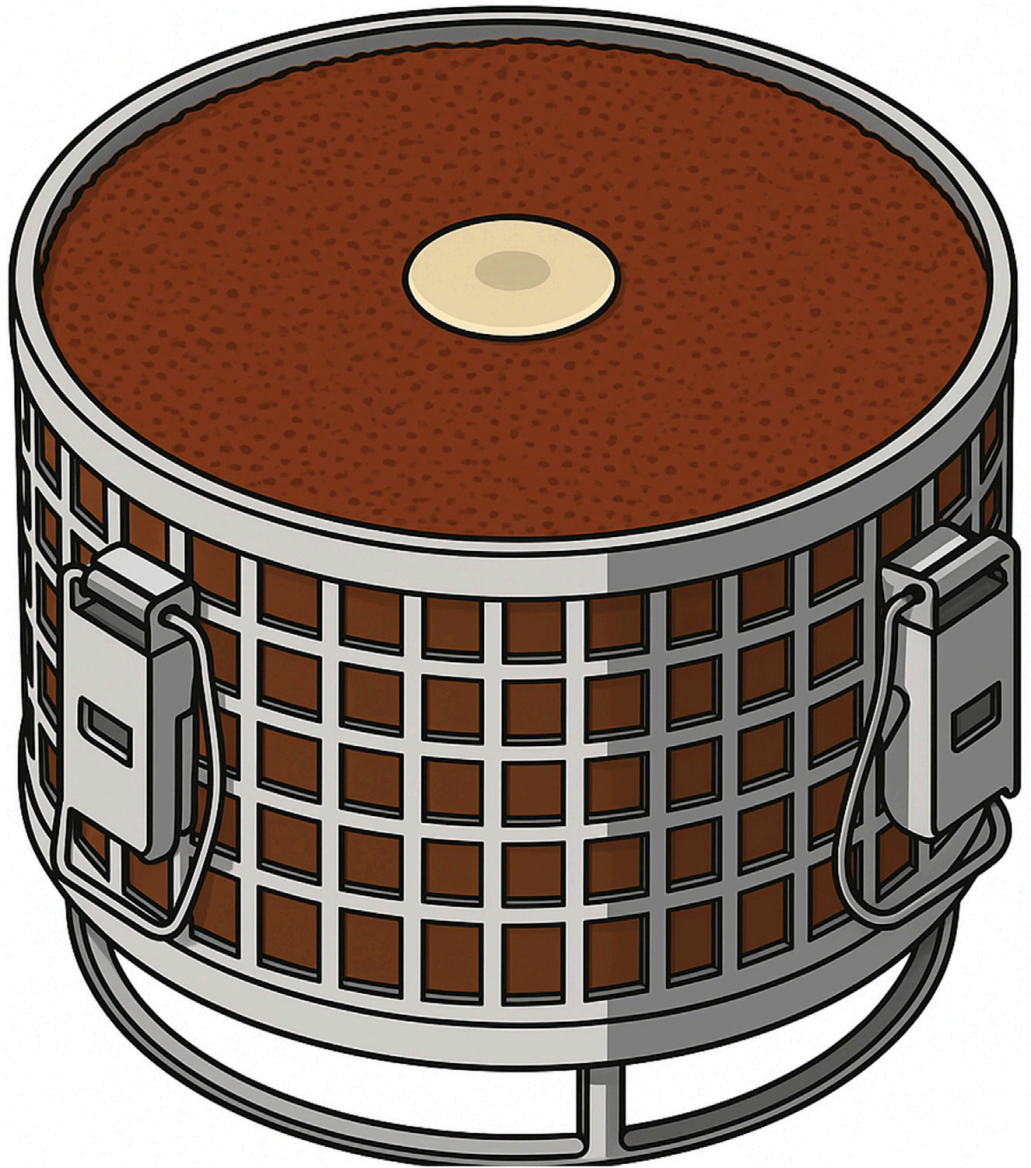
Basket with a central pin in Hardtank^®^ technology.

During the maceration process, the basket with a central pin is immersed in water. The basket features holes through which water flows in a closed circuit between the basket and the coffee bed (Figure 2).

Substances contained in the water rinse the coffee bed through a mesh, on which a natural coffee filter is formed. The fast cold extraction process allows for the precise adjustment of pressure parameters, which significantly impact the sensory quality of the resulting product. Adjusting pressure parameters contrasts with the traditional 24-h “cold brew” maceration, where coffee particles are soaked in cold water within a sealed container without additional interventions, such as stirring. This method allows for improved control over the sensory quality of the brew (Stanek et al., 2021). The technological stages of producing beverages using the Hardtank^®^ device include: receiving raw materials, grinding (conducted in a closed system directly into the extraction basket placed on a scale), and transporting the basket with ground coffee to the extraction room. Preparation for extraction involves filling the Hardtank^®^ extractor (HT1000, Hard Beans sp. z o.o., Opole, Poland) with water purified of mineral salts, screwing the extraction baskets together, and loading them into the device. Extraction lasts approximately 30 min, during which the pressure is modulated using a fluorescent display.

After extraction, the coffee is transferred via pipes to an appropriate tank. In the subsequent stage, the processes of mixing, cooling, and the addition of supplements (inulin 0.5% w/v, oat drink 5% v/v, sugar 2% w/v; 5 min at 200 rpm) are carried out successively (Fetco, 2024). During the bottling stage (330 mL), the cans are first rinsed with UVC-sterilized water, flushed with CO_2_ to adjust headspace pressure, filled with coffee and then infused with liquid nitrogen (1.2 g per can) using a CHART dispenser; the lid is immediately placed and sealed (Hard Beans, 2023).

Pasteurization is the next step, during which the product undergoes thermal stabilization at 72 °C for 15 s, monitored with a wireless probe (Fetco, 2024). The subsequent steps involve labeling, packaging, and storage in a buffer zone, where the product is quarantined until microbiological criteria are confirmed: total aerobic count (TC) ≤ 10 CFU/mL, absence of *Salmonella* and *Listeria* in 25 g, yeasts (Y) ≤ 2 CFU/mL, molds (M) < 1 CFU/mL C (Fetco, 2024; Hard Beans, 2023; Hard Beans, 2025).

The finished Nitro Cold Brew product is stored under controlled conditions (4 °C–20 °C, 35%–70% RH, relative humidity) and then released according to FIFO (First In, First Out) principles, meaning that the earliest produced batches are dispatched first to ensure proper stock rotation and quality control, before dispatch to the contractor (Hard Beans^®^ data, 2023).

### 2.4 Control infusions

Aqueous and methanol-water extracts were prepared from green and roasted coffee beans. Aqueous coffee extracts were prepared according to the SCA procedure (Fernández-Alduenda et al., 2023). Ground green coffee beans (labeled “G”) and roasted coffee beans (labeled “B”) were obtained by grinding whole beans using a Mahlkoenig VTA 6S grinder (grind setting: 5). The ground material was then accurately weighed, and six portions of 2.75 g were prepared in separate beakers for extraction. Aqueous extracts were prepared by pouring 50 mL of RO AKVO 120 ppm quality water at 93 °C over freshly ground beans. The same RO-purified water (120 ppm TDS) was used for all extractions to ensure comparability with the commercial Nitro Cold Brew beverage. Both water extractions were carried out for 5 min.

Methanolic extracts were prepared in three formats.

1. Shake extraction: 2.75 g of ground coffee was placed in a conical flask, 50 mL of 70% methanol (70% MeOH) was added at room temperature, and the mixture was shaken for 5 min at 130 rpm (shaker IST-3075R, Jeio Tech).
2. Reflux extraction (70% MeOH): 2.75 g of ground coffee was placed in a conical flask, 50 mL of 70% methanol was added, and the extract was refluxed under a condenser for 2 h at the solvent’s boiling point.
3. Reflux extraction (100% MeOH): 2.75 g of ground coffee was placed in a conical flask, 50 mL of 99.8% methanol was added, and the extract was refluxed under a condenser for 2 h at the solvent’s boiling point.

Table 1 lists the codes assigned to each experimental setup.

**TABLE 1:** Sample codes and corresponding extraction conditions.

No	Coffee type (batch)	Solvent	Extraction method	Sample code
1	Roasted coffee “B” (2022, 1)	Water (W)	Brewing (5 min)	B_W_1
2	Roasted coffee “B” (2024, 2)	Water (W)	Brewing (5 min)	B_W_2
3	Roasted coffee “B” (2022, 1)	Methanol 70%	Reflux extraction (2 h) (C)	B_M_1_70_C
4	Roasted coffee “B” (2024, 2)	Methanol 70%	Reflux extraction (2 h) (C)	B_M_2_70_C
5	Roasted coffee “B” (2024, 2)	Methanol 99.8%	Reflux extraction (2 h) (C)	B_M_2_100_C
6	Roasted coffee “B” (2024, 2)	Methanol 70%	Shake extraction (5 min, room temperature)	B_M_2_70
7	Green coffee “G” (2022, 1)	Water (W)	Brewing (5 min)	G_W_1
8	Green coffee “G” (2024, 2)	Water (W)	Brewing (5 min)	G_W_2
9	Green coffee “G” (2022, 1)	Methanol 70%	Reflux extraction (2 h) (C)	G_M_1_70_C
10	Green coffee “G” (2024, 2)	Methanol 70%	Reflux extraction (2 h) (C)	G_M_2_70_C
11	Green coffee “G” (2024, 2)	Methanol 99.8%	Reflux extraction (2 h) (C)	G_M_2_100_C
12	Green coffee “G” (2024, 2)	Methanol 70%	Shake extraction (5 min, room temperature)	G_M_2_70
13	Nitro Cold Brew “B” (2022, 1)	Water (W)	Hardtank^®^	B_W_1_HT
14	Nitro Cold Brew “B” (2024, 2)	Water (W)	Hardtank^®^	B_W_2_HT

### 2.5 Preparation of coffee beverage and control coffee extracts for testing

The water and methanol-water extracts, as well as the commercial Nitro Cold Brew coffee beverage, were filtered using paper filters (quantitative paper circles–middle, Filtrak).

### 2.6 Determination of total phenolic compound content

The TPC content in the tested macerates was determined spectrophotometrically at a wavelength of 765 nm, following a modified Singleton and Rossi method (Singleton and Rossi, 1965; Wyrostek and Kowalski, 2021). The modification involved proportional adjustments to the volume of individual chemical reagents. A 5 mL volumetric flask was filled with 0.02 mL of the test extract and 3.16 mL of water, then 0.10 mL of Folin–Ciocalteu reagent was added, mixed, and allowed to stand for 5 min. Next, 0.60 mL of a saturated sodium carbonate (Na_2_CO_3_) solution was added, the mixture was mixed again, and incubated at 40 °C for 30 min. Absorbance was measured against a blank prepared in the same way, except that 0.02 mL of water was used in place of the test extract. The results were expressed as gallic acid equivalents (GAE). The concentrations were calculated using a calibration curve prepared with gallic acid standards in the range of 10–60 mg/L (10, 20, 30, 40, 50, 60 mg/L): y = 1.0582x–0.0448, *r* ^2^ = 0.9999. Each sample was diluted appropriately to fall within the range of the standard curve. All analyses were conducted in triplicate.

### 2.7 Determination of total flavonoid content

The TFC content in the tested coffee extracts was determined spectrophotometrically at a wavelength of 510 nm, based on a modified procedure by Karadeniz et al. (2005) (Wyrostek and Kowalski, 2021). A 1 mL portion of the infusion was measured into a 10 mL volumetric flask, followed by the addition of 5 mL of redistilled water and 0.3 mL of 5% (w/w) aqueous sodium nitrate solution. The mixture was left to stand for 5 min, after which 0.6 mL of 10% (w/w) aqueous aluminum chloride hexahydrate solution was added and mixed again. After another 5 min, 2 mL of 1M aqueous NaOH solution was added, and the volume was adjusted to the mark with redistilled water. The absorbance of the samples prepared in this manner was measured at 510 nm against a blank. Results were expressed as epicatechin equivalents (EE) and calculated using a calibration curve prepared with epicatechin standards in the concentration range 10–400 mg/L (10; 50; 100; 150; 200; 250; 300; 400 mg/L): y = 0.002x–0.008, *r* ^2^ = 0.9990. Each sample was diluted to fall within the range of the standard curve. All analyses were conducted in triplicate.

### 2.8 Analysis of CAF and selected phenolic acids

CAF and selected phenolic acids (ChA, CA, p-coumaric acid pCA) were analyzed using reversed-phase high-performance liquid chromatography (RP-HPLC) with modifications for flow rate and mobile phase composition (Wołosiak et al., 2007). A spectrophotometric diode-array detector (DAD) was used for analyte detection. Concentrations of the analytes in the extracts were determined using calibration curves derived from standard solutions (0.5; 2.75; 8.25; 13.75; 27.50; 41.25; 55.00 mg/L); CAF: y = 1.5029x + 0.1803, *r* ^2^ = 1.0000; ChA: y = 1.417x + 0.1427, *r* ^2^ = 0.9999, CA: y = 2.6824x+0.7049, *r* ^2^ = 0.9999; pCA: y = 3.759x + 0.4989, *r* ^2^ = 0.9999. Identification was based on retention time and the UV spectrum of reference standards.

**FIGURE 3:**
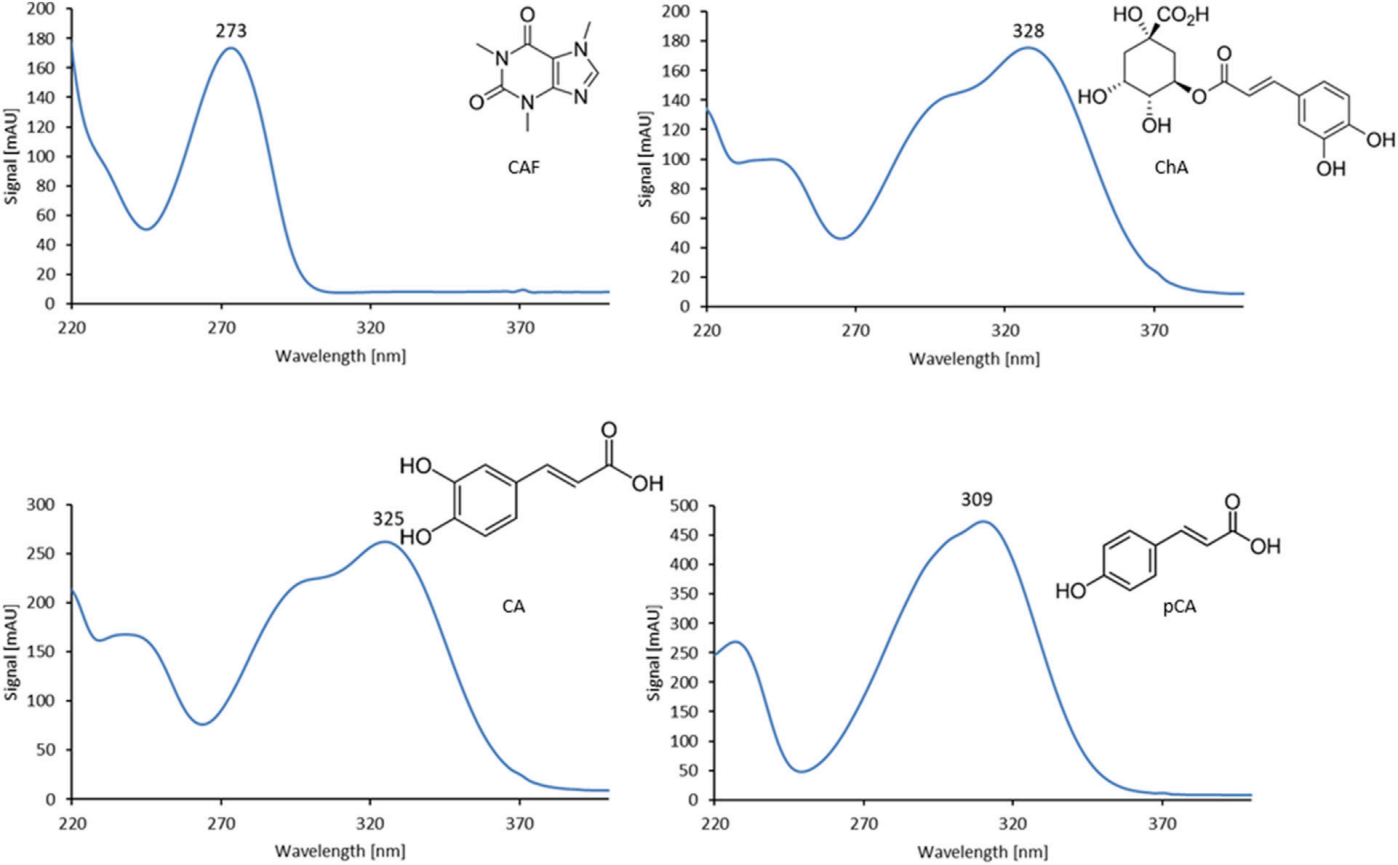
UV spectra of CAF, ChA, CA, and pCA (220–400 nm).

The analyses were conducted using a Varian HPLC system (Palo Alto, CA, United States) equipped with a DAD (type 335), pump (type 210), Rheodyne 7725i dosing valve, column thermostat, and a Gemini 150 × 4.6 mm (3 µm C18) chromatographic column with a Gemini C18 4 × 3 mm pre-column (Phenomenex, Torrance, CA, United States). The injection volume was 20 µL. The mobile phase was methanol (A) and a solution of orthophosphoric acid adjusted to pH 2.8 (B), pumped at the rate of 0.6 mL/min. Gradient elution was applied: 0.0 min: A = 20%, B = 80%; 10.0 min: A = 50%, B = 50%; 15.0 min: A = 70%, B = 30%; 20.0 min: A = 100%, B = 0%; 22.0 min: A = 100%, B = 0%; 24.0 min: A = 20%, B = 80%. Data were collected over a wavelength range of 190–400 nm at 30 °C, while quantitative analysis of individual compounds was performed at the characteristic wavelength for each analyte (ChA–328 nm; CA–325 nm; pCA–309 nm; CAF–273 nm). The specific wavelengths were selected based on the maximum absorbance (λmax) determined from the UV spectra of each standard compound (Figure 3), ensuring the highest sensitivity and selectivity of detection.

**FIGURE 4:**
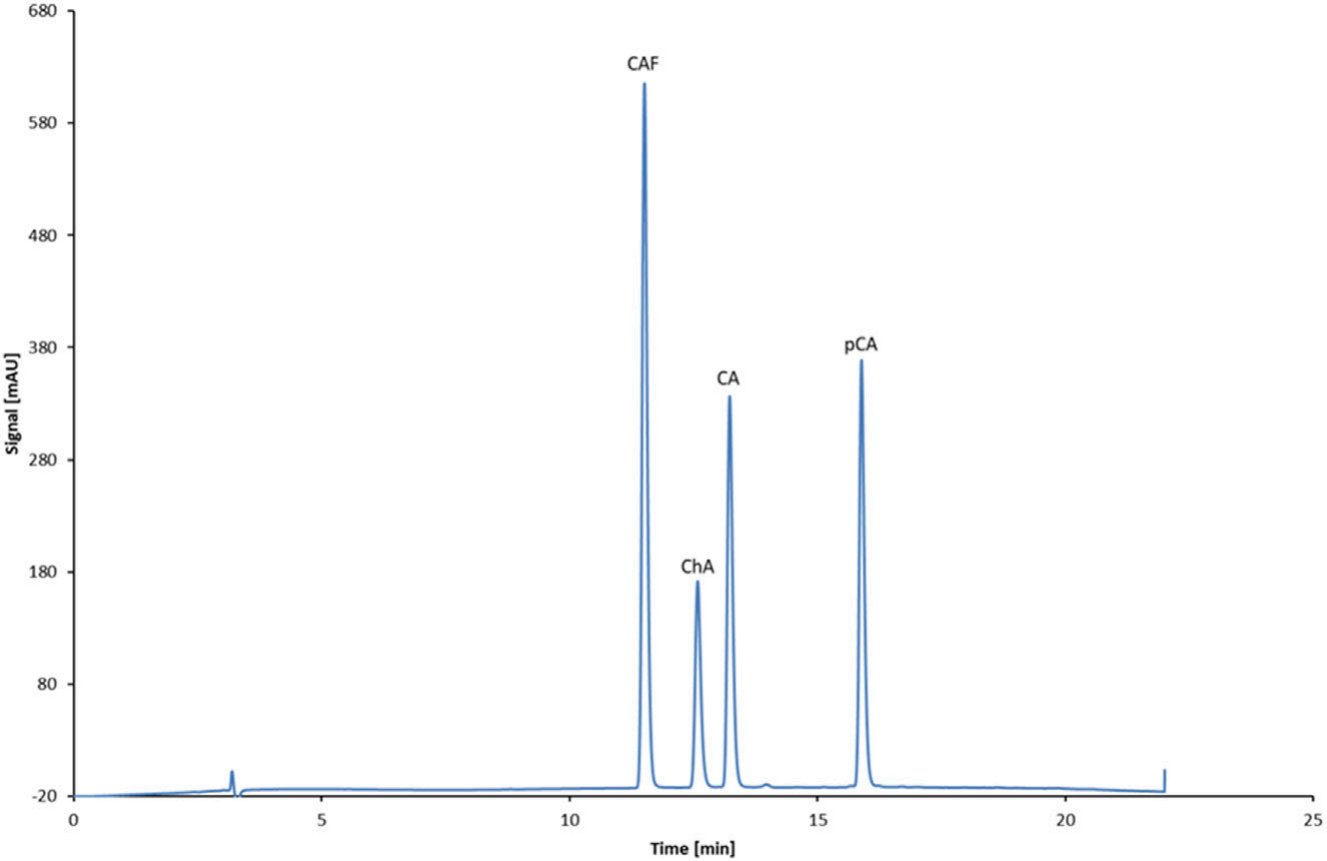
Chromatogram showing the separation of standards: CAF, ChA, CA, and pCA.

Figure 4 shows the chromatogram of the separation of standards (CAF and selected phenolic acids).

In addition, derivatives of CA and ChA were identified based on their UV spectra, which were compared to those of CA and ChA (correlation above 97%). This approach was informed by literature data (Jeszka-Skowron et al., 2022), indicating the presence of numerous isomers, including 5-O-caffeoylquinic acid, 4-O-caffeoylquinic acid, and 3,4-dicaffeoylquinic acid, among others, in coffee products. Quantitative analysis of ChAd and CAd was performed using calibration curves for ChA and CA, respectively, assuming identical detector response (concentration–absorbance) for the pairs ChA–ChAd and CA–CAd.

Coffee extracts were diluted appropriately (5-fold and 10-fold), centrifuged (5 min, 4,500 rpm), filtered (1–2 mL) using a syringe filter (0.45 µm diameter), and 20 µL of each solution was injected into the chromatographic system.

### 2.9 Extraction efficiency

Extraction efficiency was calculated as the percentage of biologically active compounds in the extract relative to their content in the methanol-water reference extract. The extraction efficiency of the 70% methanol reflux method was arbitrarily set at 100% as a methodological reference for comparing all tested extracts. The extraction efficiency for water infusions prepared by the classical method and the Hardtank^®^ method was calculated using the Formula 1:

$$Y\left[\%\right]=\frac{C_{W/HT}\times 100\%}{C_{MeOH}}$$ (1)

where:

*C*_*MeOH*_–concentration of the component in the 70% methanol extract [mg/100 mL]

*C*_*W/H*T_–concentration of the component in water extracts prepared by the classical method (W) or Hardtank^®^ method (HT) [mg/100 mL]

### 2.10 Free radical-scavenging ability by the use of a stable DPPH radical

Antioxidant activity was assessed using a modified Brand-Williams method with the synthetic radical DPPH (2,2-diphenyl-1-picrylhydrazyl) dissolved in ethanol (Brand-Williams et al., 1995; Wyrostek and Kowalski, 2021). Absorbance was measured at 517 nm. The DPPH solution was adjusted to an absorbance of approximately 0.9 at 517 nm and stored in the dark until use. Each test sample consisted of 1.5 mL of the DPPH solution and 20 µL of the coffee extracts. Absorbance (A) was recorded 30 min after initiating the reaction. Inhibition of the DPPH radical was calculated using the Formula 2:

$$I\left[\%\right]=\frac{100\times \left(A_{0}-A_{1}\right)}{A_{0}}$$ (2)

where:

*A*_*0*_–absorbance of the control

*A*_*1*_–absorbance of the sample

The antioxidant activity results were expressed as Trolox equivalents (TE) by constructing linear calibration curves for nine Trolox concentrations ranging from 0.2 to 2.0 mM (0.2; 0.4; 0.6; 0.8; 1.0; 1.2; 1.4; 1.6; 1.8; 2.0 mM): y = 24.782x‒2.541, *r* ^2^ = 0.9997, following the procedure described by Rumpf et al. (2023). Each sample was diluted appropriately to the range of a calibration curve prepared with Trolox standards. All measurements were performed in triplicate.

### 2.11 Ferric reducing antioxidant power (FRAP) assay

The total antioxidant potential of a sample was determined using the ferric reducing ability of plasma (FRAP) assay (Benzie and Strain, 1999) as a measure of antioxidant power. Briefly, the FRAP reagent was prepared immediately before use by mixing: acetate buffer (300 mM, pH 3.6), a solution of 10 mM 2,4,6-tripyridyl-s-triazine (TPTZ) in 40 mM HCl, and iron (III) chloride hexahydrate solution (20 mM FeCl_3_·6H_2_O) in a volumetric ratio of 10:1:1 (v/v/v). For each determination, 3,000 µL of FRAP reagent and 100 µL of sample (brew solution) were mixed and incubated; absorbance was read at 593 nm after 180 min. A standard curve was prepared from fresh stock solutions of iron (II) sulfate heptahydrate (FeSO_4_·7H_2_O) at concentrations of 100–1,000 mM (100; 200; 300; 500; 700; 900; 1,000 mM). All solutions and standards were prepared fresh on the day of use. FRAP values were converted to TE by constructing linear calibration curves with nine Trolox standards in the range 0.05–0.5 mM (0.05; 0.10; 0.15; 0.20; 0.25; 0.30; 0.35; 0.40; 0.45; 0.50 mM): y = 1.6043x+0.056, *r* ^2^ = 0.9967, following the procedure described by Rumpf et al. (2023). Each sample was diluted as necessary to fall within the Trolox calibration range. All measurements were performed in triplicate.

### 2.12 Statistical analysis

Data were analyzed using one-way ANOVA followed by Duncan’s test in the SAS statistical system (SAS version 9.1, SAS Institute, Cary, NC, United States). Statistical significance was set at p ≤ 0.05. Data are presented as mean ± standard deviation (SD) from three replicates. Different letters (a, b, c, etc.) denote statistically significant differences at p < 0.05.

## 3 Results and discussion

### 3.1 Total phenolic and flavonoid concentration

**FIGURE 5:**
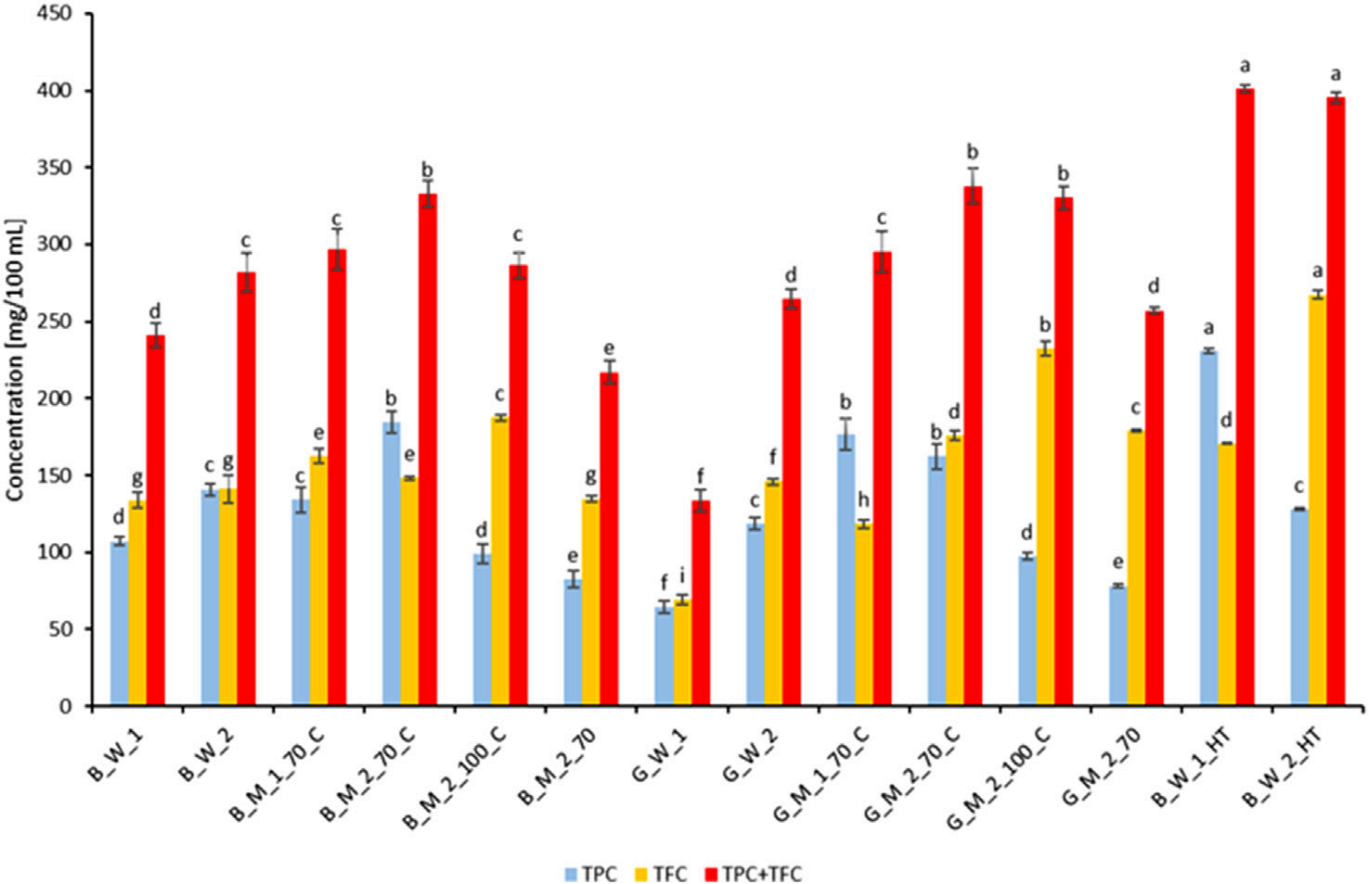
Concentrations of TPC and TFC in experimental extracts of roasted and green coffee and in the commercial Nitro Cold Brew beverage (HT). Sample codes as in Table 1. Different letters (a, b, c …) indicate significant differences at p < 0.05.

Figure 5 presents the concentrations of TPC and TFC in the experimental extracts of roasted and green coffee, as well as in the commercial Nitro Cold Brew beverage (HT) obtained using the HT extraction method, as described in Table 1.

In the traditionally prepared extracts (water infusion, 70% and 100% methanol reflux, 70% methanol shake), TPC and TFC were as follows. The lowest TPC (64.5 ± 4.0 mg/100 mL) and TFC (68.9 ± 3.1 mg/100 mL) were observed in the green coffee water infusion (G_W_1), whereas the highest values occurred in the reflux methanol extracts: B_M_2_70_C exhibited TPC = 184.7 ± 7.2 mg/100 mL and TFC = 148.3 ± 1.2 mg/100 mL, and G_M_2_100_C showed TPC = 97.7 ± 2.3 mg/100 mL and TFC = 232.6 ± 4.9 mg/100 mL. The 70% methanol shake extraction (B_M_2_70) yielded only 82.4 ± 5.7 mg/100 mL TPC and 134.7 ± 1.9 mg/100 mL TFC, demonstrating that both extraction time (5 min vs. 2 h) and the presence of a condenser are critical for efficient polyphenol release.

In the HT extracts (Nitro Cold Brew), intermediate TPC values were obtained—230.8 ± 1.7 mg/100 mL for B_W_1_HT and 128.0 ± 0.9 mg/100 mL for B_W_2_HT—while maintaining high flavonoid levels: 170.6 ± 0.5 mg/100 mL (B_W_1_HT) and 267.4 ± 2.5 mg/100 mL (B_W_2_HT). The use of Hardtank^®^ technology in Nitro Cold Brew allowed for a combined TPC + TFC concentration of 401.3 ± 2.2 mg/100 mL and 395.4 ± 3.4 mg/100 mL, respectively—significantly higher than those in shake-extracted methanolic extracts of roasted and green coffee (217–257 mg/100 mL) and water infusions of both bean types (133–282 mg/100 mL). The phytochemical differences observed between HT extracts from roasted beans harvested in 2022 and 2024 may reflect qualitative and quantitative variations in phenolic profiles driven by agronomic and climatic factors; nevertheless, HT technology consistently produced a beverage with a high total TPC + TFC content. These results confirm that the Hardtank^®^ process, by combining dynamic flow, controlled pressure, and a short extraction time (30–50 min), promotes efficient recovery of phenolic compounds. Unlike classical cold brew infusions (24 h) or reflux extractions, HT not only matches traditional methods in TFC yield but markedly enhances the overall phenolic potential, likely due to the hydrodynamic conditioning of the coffee bed, which minimizes concentration gradients and ensures continuous solvent–particle contact (Zhang et al., 2022a).

In traditional water infusions of roasted coffee (B_W_1, B_W_2) and methanol–water extracts (M_70), we observed TPC values of 107.1 ± 2.9 and 141.0 ± 3.9 mg/100 mL in the water infusions, and 134.0 ± 8.2 and 184.7 ± 7.2 mg/100 mL in the extracts B_M_2_70 and G_M_2_70, respectively (mean differences ≈27 mg/100 mL). Ferruzzi (2010) reported that the TPC in traditional coffee infusions could reach up to 169 mg/100 mL. Literature data indicate comparable efficiencies of methanol and water extractions for total phenolic fractions, as observed in studies on *Clinopodium vulgare* (Sarikurkcu et al., 2015) and coffee pulp (Hu et al., 2023). Other studies have reported TPC values in roasted coffee infusions ranging from 88.41 mg/100 mL to 485.50 mg/100 mL (Muñoz et al., 2020). In the classic water infusion of green coffee (G_W_1), we recorded a TPC of 64.5 ± 4.0 mg/100 mL, which is over 3.5 times lower than that of the Nitro Cold Brew beverage (HT; 230.8 ± 1.7 mg/100 mL in B_W_1_HT) and 2.7 times lower than in the green coffee methanol–water extract (G_M_1_70_C; 176.9 ± 10.2 mg/100 mL).

Literature reports TPC in green coffee extracts reaching 60.26 mg/mL (6,026 mg/100 mL) when ten times more raw material was used (Silva et al., 2022), as well as values ranging from 99 to 352.74 mg/100 mL under identical water extraction conditions (Muñoz et al., 2020). In our study, the highest TPC in the HT beverage was 2.2-fold greater than in traditional roasted coffee infusions (2022 harvest) and 1.6-fold greater than in B_M_2_70 methanol–water extracts of roasted beans (2024 harvest). Relative to green coffee methanol–water extracts (G_M_2_70; 2024 harvest), the HT beverage achieved a 1.6-fold higher polyphenol concentration. Previous reports on cold brew (18 h, 22 °C) indicated TPC of approximately 258 mg/100 mL (Zhang et al., 2022a), consistent with our observations.

In traditional water infusions of roasted coffee, TFC was found to range from 133.7 to 141.1 mg QE/100 mL, substantially exceeding literature values of 7.12–10.40 mg QE/200 mL (3.5–5.2 mg QE/100 mL) reported for brews prepared in copper vessels at 90 °C with a 5 g/200 mL ratio (Pintać et al., 2022). In drip brew (10 g/200 mL, 4 min), Santanatoglia et al. (2023), delivered 18.7 ± 0.9 mg QE/100 mL, while Morais et al. (2023), under similar conditions (10 g/200 mL, 5 min), obtained 16.3–24.8 mg QE/100 mL. In model comparisons of cold vs. hot brew (10 g/200 mL; 6 h, 4 °C vs. 6 min, 96 °C), Muzykiewicz-Szymańska et al. (2021) observed a TFC range of 69.8–1,007.7 mg/100 mL, and Schwarzmann et al. (2022), using 20 g/200 mL, reported as much as 900 mg rutin equivalents per 100 mL (9 g/L). Morais et al. (2023) further reported 194–358.8 mg ECE/100 mL in Arabica and Robusta capsule brews and Mahalingam and Zaidi (2024). Measured 298–890 mg CE/100 mL in 70% MeOH Soxhlet extracts. In our 70% MeOH extracts of roasted coffee (2024 harvest), TFC ranged from 134.7 mg/100 mL (B_M_2_70) to 148.3 mg/100 mL (B_M_2_70_C), and reached 162.5 mg/100 mL in B_M_1_70_C (2022 harvest). Green coffee extracts yielded TFC of 118.4 mg/100 mL (G_M_1_70_C, 2022 harvest) to 175.7 mg/100 mL (G_M_2_70_C) and 179.0 mg/100 mL (G_M_2_70, 2024 harvest). By comparison, Nitro Cold Brew (HT) contained 170.6 mg/100 mL (B_W_1_HT) and 267.4 mg/100 mL (B_W_2_HT), representing 1.2–1.9× higher TFC than traditional water infusions and 1.1–1.8× higher than 70% MeOH extracts. Notably, 100% MeOH reflux extractions yielded TFC of 187.5 mg/100 mL (B_M_2_100_C) and 232.6 mg/100 mL (G_M_2_100_C, 2024 harvest), both significantly lower than the 267.4 mg/100 mL achieved by HT in B_W_2_HT (2024 harvest). Previously published studies have reported lower flavonoid concentrations in green coffee beans (Hečimović et al., 2011) while roasting coffee beans is associated with increased flavonoid content, likely due to Maillard reactions (Król et al., 2020). However, other studies have noted higher flavonoid levels in green coffee beans compared to roasted beans (Hudáková et al., 2016).

It should also be noted that a pure alcoholic solvent is more effective for flavonoid extraction than aqueous mixtures. Kowalska et al. (2021), demonstrated that absolute ethanol extracted higher levels of TFC from mint and nettle leaves than 50% ethanol. Conversely, those authors observed that 50% ethanol–water mixtures yielded greater total phenolic contents (TPC) from the same plant materials than pure ethanol.

The 70% methanol reflux extraction merits particular attention: in B_M_1_70_C and B_M_2_70_C, TPC values of 134.0 ± 8.2 and 184.7 ± 7.2 mg/100 mL and TFC values of 162.6 ± 5.0 and 148.3 ± 1.2 mg/100 mL were obtained, respectively. These combined TPC + TFC concentrations exceeded those in classical water infusions but were lower than those achieved by HT in total phenolic potential. Green coffee extracts G_M_1_70_C and G_M_2_70_C yielded 176.9 ± 10.2 and 162.2 ± 8.4 mg/100 mL TPC and 118.4 ± 3.0 and 175.7 ± 3.1 mg/100 mL TFC, underscoring the high efficiency of methanol–water (70%) with full condenser reflux. These results confirm that the presence of 30% water increases solvent polarity, facilitating the solubilization of a broad spectrum of phenolics, particularly polar species. The addition of water to methanol or ethanol optimizes phenolic solubility (Hu et al., 2023; Kowalska et al., 2021; Sarikurkcu et al., 2015), and reflux conditions further enhance extraction efficiency.

In summary, 70% methanol–water reflux extraction demonstrated high recoveries of both TPC and TFC, providing a robust reference for comparative analyses; however, Hardtank^®^ technology combines rapid processing with the highest overall polyphenolic yield, making it a promising method for the production of functional coffee beverages.

### 3.2 Concentrations of CAF and selected phenolic acids

**FIGURE 6:**
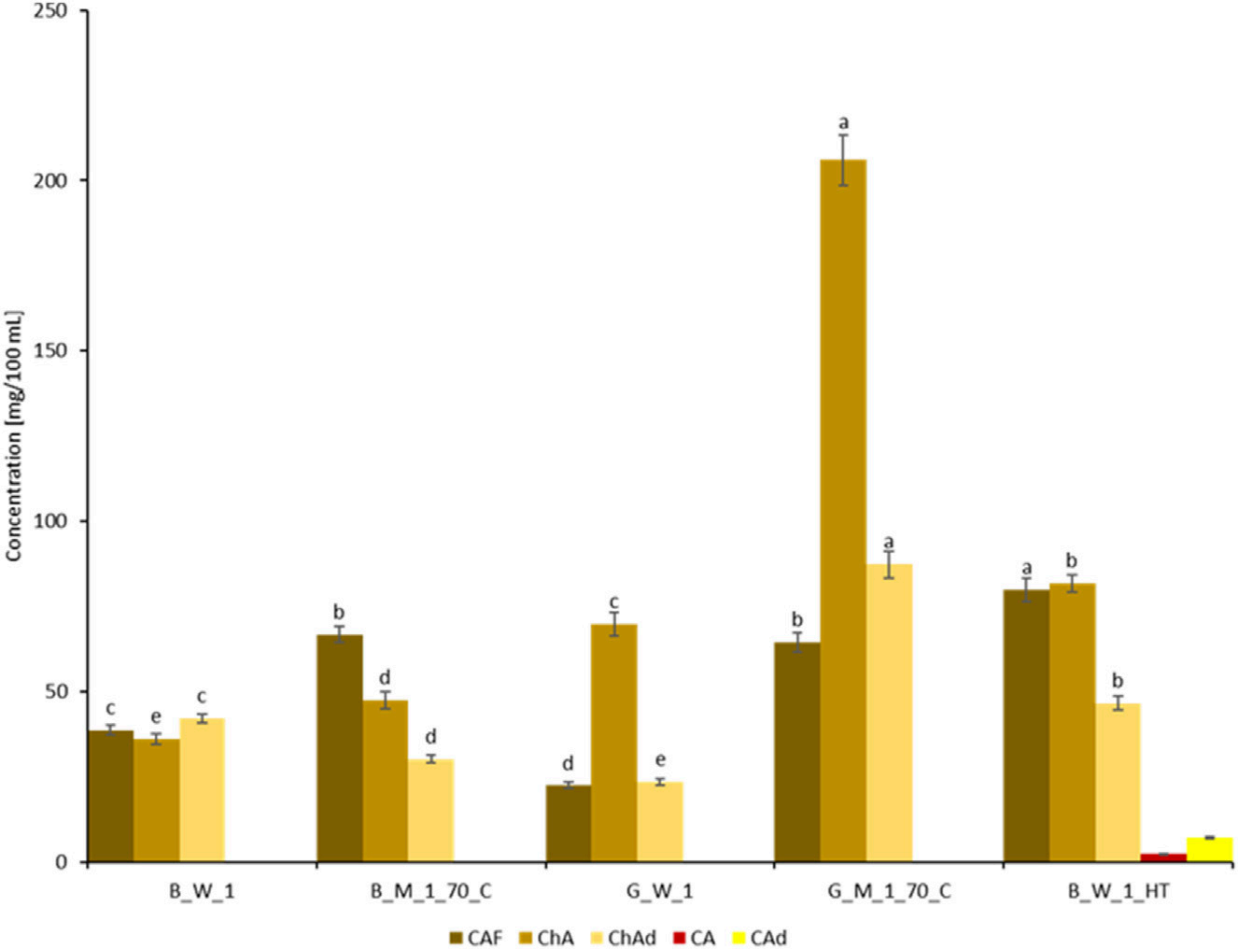
Concentrations of CAF, ChA, ChAd, CA, and CAd in experimental extracts of roasted and green coffee and in the commercial Nitro Cold Brew beverage (HT). Sample codes as in Table 1. Different letters **(a–e)** indicate significant differences at p < 0.05.

**FIGURE 7:**
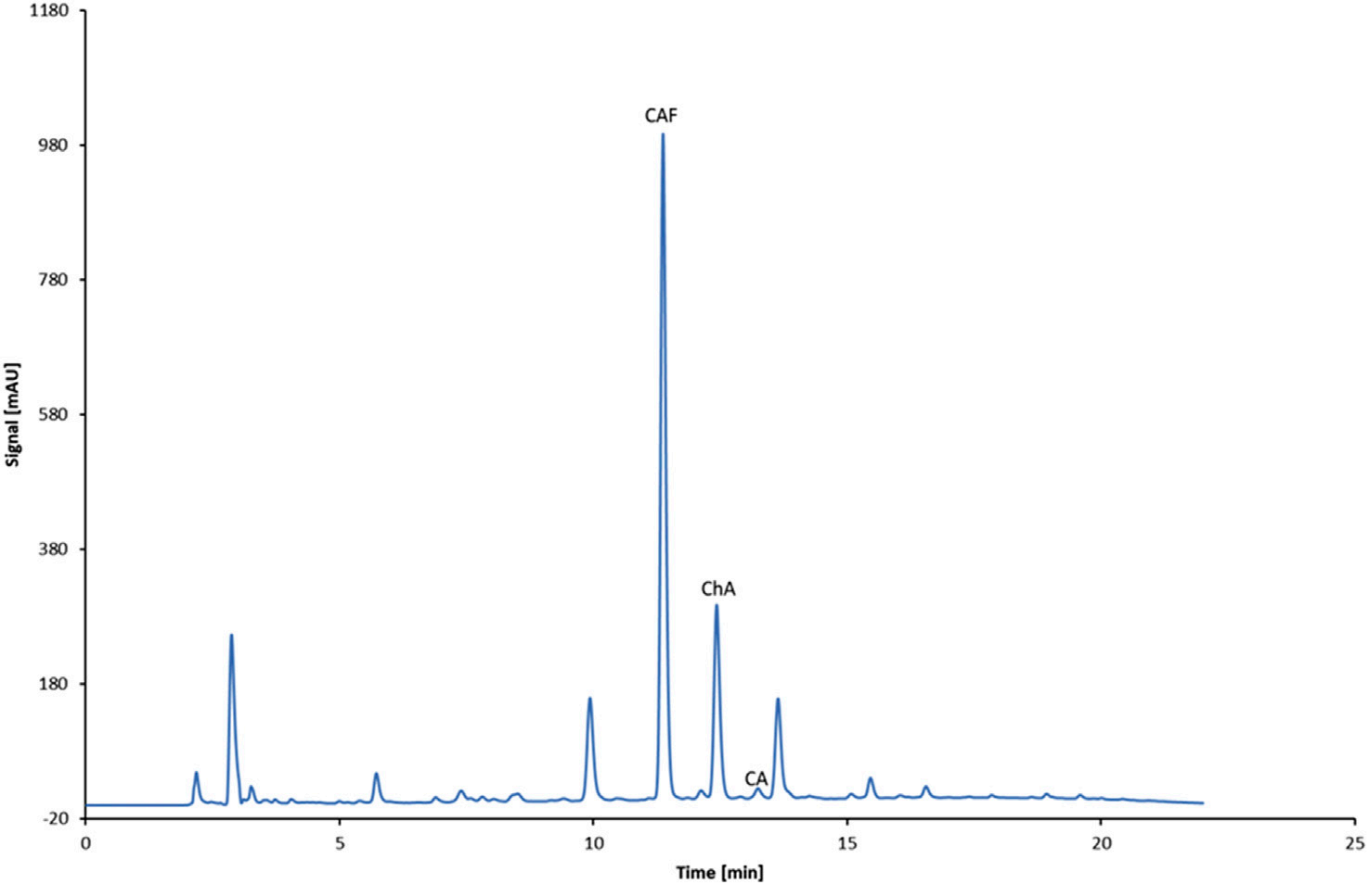
Representative chromatogram of compounds (CAF, ChA, and CA) identified in the commercial Nitro Cold Brew beverage (B_W_1_HT).

Figure 6 presents the concentrations of CAF, ChA, ChAd, CA, and CAd in experimental extracts of roasted and green coffee and in the commercial Nitro Cold Brew beverage, obtained from beans harvested in 2022. An example chromatogram from the separation of identified components in the commercial Nitro Cold Brew beverage is presented in Figure 7.

The conducted study revealed that the Nitro Cold Brew beverage (B_W_1_HT) exhibited the highest CAF concentration (79.8 mg/100 mL), while the lowest concentration was recorded in the green coffee infusion G_W_1 (22.4 mg/100 mL). Furthermore, methanol-water coffee extracts demonstrated higher CAF concentrations than the tested coffee infusions. For instance, the CAF content in the classic roasted coffee infusion B_W_1 was nearly two times lower than in the methanol-water extracts of roasted coffee B_M_1_70_C (38.7 mg/100 mL vs. 66.7 mg/100 mL). Literature reports confirm similar CAF concentrations in traditional roasted coffee infusions, with the “Americano” brewing method yielding an average CAF content of 43.7 mg/100 mL (Santini et al., 2011). The CAF concentration in methanol-water extracts of green coffee G_M_1_70_C was three times higher than in the green coffee water infusion G_W_1 (64.4 mg/100 mL vs. 22.4 mg/100 mL). Literature data indicate that CAF concentrations in coffee infusions can range widely, from 1.4 mg/100 mL to 8.8 mg/100 mL, depending on the preparation method (Macheiner et al., 2019). The CAF content in the Nitro Cold Brew beverage (B_W_1_HT) was approximately 80 mg/100 mL, indicating more efficient CAF extraction during the tested technological process compared to the traditional roasting and brewing conditions used in the experiment (approximately two times higher CAF content) and compared to methanol-water extraction (four times higher CAF content). In a separate study, researchers found a comparable CAF concentration (on average, 97 mg/100 mL) in cold brew beverages prepared from roasted Arabica coffee beans, although the extraction time in the cited study was 6 h, compared to 30 min in our study (Bellumori et al., 2021). Other reports documented a CAF concentration of od 48.87 mg/100 mL do 60.60 mg/100 mL in cold brew beverages prepared with an 9-h extraction time at 4 °C i od 54.01 mg/100 mL do 78.30 mg/100 mL in cold brew beverages prepared with an 24-h extraction time at 4 °C (Muzykiewicz-Szymańska et al., 2021). In contrast, Zhang et al. (2022a) reported that the CAF concentration in cold brew beverages prepared with an 18-h extraction at 22 °C was 48 mg/100 mL. Additionally, another study noted a CAF content of 125 mg/100 mL in cold brew beverages prepared from green coffee beans (Macheiner et al., 2019). Given consumer preferences for coffee beverages with stimulating properties and high CAF concentrations (Agunbiade et al., 2022), the Nitro Cold Brew (HT) extraction method offers an appealing alternative to traditional coffee infusions.

The highest ChA concentration was observed in methanol-water extracts of green coffee_M_1_70_C (approximately 206.1 mg/100 mL), whereas the lowest concentration was found in the classic roasted coffee infusion B_W_1 (36.1 mg/100 mL). Methanol-water extracts of green coffee G_M_1_70_C also exhibited the highest concentration of ChAd (87.4 mg/100 mL), while the lowest concentration was noted in methanol-water extracts of roasted coffee G_W_1 (23.5 mg/100 mL).

The concentration of ChA in the traditional water infusion of roasted coffee B_W_1 and methanol-water extract B_M_1_70_C was within a similar range (36.1 mg/100 mL vs. 47.5 mg/100 mL). These findings align with prior reports documenting ChA concentrations in coffee infusions from seeds with varying degrees of roasting: 42.3 mg/100 mL for dark-roasted seeds, 79.2 mg/100 mL for medium-roasted seeds, and 120.7 mg/100 mL for light-roasted seeds (Rune et al., 2023). In another study, the authors demonstrated that the ChA content ranged from 0.412 to 2.262 mg/100 mL (Muñoz et al., 2020). The ChA content in the Nitro Cold Brew beverage (B_W_1_HT) was approximately 81.8 mg/100 mL, more than twice the concentration found in traditional roasted coffee infusions and approximately 10 mg/100 mL higher than in green coffee infusions G_W_1. Zhang et al. (2022a) reported similar ChA concentrations in cold brew extracts, averaging 96 mg/100 mL, with an 18-h extraction time at 22 °C. Another study reported ChA concentrations of up to 290 mg/100 mL in cold brew extracts obtained with a 6-h extraction time (Bellumori et al., 2021). The cited authors observed a wide range of concentrations in the obtained extracts, ranging from 112 mg/100 mL to 390 mg/100 mL (Bellumori et al., 2021). The variability in ChA concentrations may result from its sensitivity to high temperatures during the roasting process, unlike CAF, which remains more stable. Our findings confirm a reduction in ChA content in roasted coffee extracts compared to green coffee extracts. Published studies indicate that technological processes, such as pasteurization (65 °C for 30 min), do not significantly reduce ChA concentrations (Bellumori et al., 2021). Additionally, beverages obtained via the cold brew method from green coffee were reported to contain ChA concentrations of approximately 284 mg/100 mL, comparable to those in roasted coffee extracts (Macheiner et al., 2019). Other studies noted ChA concentrations in green coffee extracts ranging from 62.8 mg/100 mL to 104 mg/100 mL (Macheiner et al., 2019) and from 0.145 mg/100 mL to 14.975 mg/100 mL (Muñoz et al., 2020). These data also confirm that the origin of coffee beans significantly influences ChA content.

The chlorogenic acid derivative content in the Nitro Cold Brew beverage (B_W_1_HT) was similar to that in traditional roasted coffee infusions B_W_1 (46.6 mg/100 mL vs. 42.0 mg/100 mL) but higher than in methanol-water extracts of roasted coffee B_M_1_70_C (30.2 mg/100 mL). Literature suggests higher extraction efficiency for ChAd (5-O-caffeoylquinic acid, 4-O-caffeoylquinic acid, 3-O-caffeoylquinic acid) using traditional brewing methods for medium-roasted coffee (114.35 mg/100 mL) and dark-roasted coffee (75.69 mg/100 mL) (Turan Ayseli et al., 2021). The highest concentration of ChAd (87 mg/100 mL) was found in methanol-water extracts of green coffee (Turan Ayseli et al., 2021). The highest concentration of ChAd was found in the methanol-water extract from green coffee G_M_1_70_C, amounting to 87.4 mg/100 mL. Previously published studies showed that the content of ChAd (5-O-caffeoylquinic acid, 4-O-caffeoylquinic acid, 3-O-caffeoylquinic acid) was higher in espresso infusions made from green coffee, reaching 106 mg/100 mL (Macheiner et al., 2019).

The presence of CA was unique to the Nitro Cold Brew beverage B_W_1_HT (2.2 mg/100 mL) and was not detected in any other tested coffee extracts or infusions. Similarly, CAd (7.1 mg/100 mL) were only found in the Nitro Cold Brew beverage. Literature data confirm low CA concentrations in coffee infusions, ranging from 1.1 mg/100 mL to 9.36 mg/100 mL for roasted coffee and from 0.59 mg/100 mL to 6.03 mg/100 mL for green coffee (Muñoz et al., 2020). Additionally, our study did not detect pCA in any of the tested coffee extracts. However, literature data report pCA concentrations ranging from 0.517 mg/100 mL to 6.583 mg/100 mL in green coffee infusions and similar levels in roasted coffee infusions (Muñoz et al., 2020).

### 3.3 Extraction efficiency

Table 2 presents the extraction efficiency of Hardtank^®^ (commercial product Nitro Cold Brew) compared to the classic coffee brewing method (for concentrations of TPC, TFC, CAF, ChA, and ChAd). The extract obtained using a 70% methanol-water mixture (B_M_1_70_C and G_M_1_70_C) was used as a reference, assuming a value of 100% as a relative value, enabling comparison of the tested extracts. The study demonstrated that Hardtank^®^ extraction (B_W_1_HT) achieved the highest efficiency for the analyzed chemical groups compared to the control extractions: 172.29% (ChA) > 165.37% (ChA + ChAd) > 154.49% (ChAd) > 135.33% (TPC + TFC) > 119.72% (CAF). The extraction efficiency of the combined TPC and TFC for Hardtank^®^ extraction from roasted beans harvested in 2024 (B_W_2_HT) was slightly lower, at 118.73%. For the control roasted coffee infusion (B_W_1), only the extraction efficiency of ChAd and the sum of ChA + ChAd exceeded 100%, reaching 139.33% and 100.72%, respectively. On the other hand, the classic green coffee extraction showed lower efficiency compared to the methanol-water extraction. Considering the goal of producing a coffee beverage with optimal concentrations of biologically active compounds, the study confirms that HT extraction enables the production of a product with the highest levels of tested active ingredients compared to traditional brewing methods.

**TABLE 2:** Extraction efficiency of individual chemical fractions across experimental setups. Codes of samples according to Table 1.

Coffee extract	Extraction efficiency [%]				
	TPC + TFC	CAF	ChA	ChAd	ChA + ChAd
B_W_1	81.19	58.07	76.17	139.33	100.72
B_W_2	84.71	nt	nt	nt	nt
G_W_1	45.16	34.85	33.88	26.87	31.79
G_W_2	78.29	nt	nt	nt	nt
B_W_1_HT	135.33	119.72	172.29	154.49	165.37
B_W_2_HT	118.73	nt	nt	nt	nt

Explanations: Total phenolic compounds (TPC), total flavonoids (TFC), caffeine (CAF), chlorogenic acid (ChA), chlorogenic acid derivatives (ChAd), water extracts (W), roasted coffee (B), green coffee (G), and the commercial Nitro Cold Brew beverage (HT), not tested (nt).

Extraction efficiency is influenced by numerous process parameters, including temperature, solvent volume, flow rate, and particle size. Methanol and ethanol are the most commonly used solvents (Amr and Al-Tamimi, 2007), with methanol being more efficient in many cases (Kapasakalidis et al., 2006). For example, studies have shown that the extraction of anthocyanins from grapes using methanol is 20% more effective than with ethanol and 73% more effective than water-based extraction. Typically, extraction procedures are sequential, systematically releasing phenolic compounds in their respective forms. Methanol and ethanol are commonly employed for exhaustive extractions aimed at isolating all biologically active compounds from plant material. Some studies suggest ethanol is more effective (Eghdami et al., 2013; Yilmaz and Toledo, 2006), while others favor methanol (Abdul Qadir et al., 2017; Lapornik et al., 2005; Mohammedi and Atik, 2011; Rahimi et al., 2018); still, there are reports indicating no significant differences between the two solvents (Siddhuraju and Becker, 2003). An important factor in selecting an extraction system for biologically active compounds is achieving the optimal ratio between different solvents, often mixed with water. Polar solvents such as methanol and ethanol, either pure or as aqueous mixtures, are widely recommended for the extraction of phenolic antioxidants from plant materials (Arabshahi-Delouee and Urooj, 2007; Peschel et al., 2006). Using an alcohol-water mixture offers the advantage of modulating solvent polarity, while the solubility of polyphenols primarily depends on the presence of hydroxyl groups, molecular size, and hydrocarbon chain length.

Extraction efficiency is determined not only by the choice of solvent but also by the solvent volume, flow rate (or static maceration), and coffee particle size. Literature reports that cold brew (10 g/200 mL, 18 h, 4 °C) and hot brew (3 min, 96 °C) are typically prepared using the same grind size, although the absence of flow significantly prolongs the time required to achieve comparable antioxidant activity (Rao and Fuller, 2018). An alternative is high-pressure processing (HPP; 400 MPa, 5–10 min), which, in standard cold brew (10 g/200 mL, 24 h, room temperature), can reduce extraction time from hours to minutes while maintaining or slightly increasing the overall extraction yield at a 1:20 ratio (Schwarzmann et al., 2022). In the drip brew method, flow rate and particle size influence extraction kinetics—fine grinding (<500 μm) generally enhances polyphenol yield, whereas excessively fast or slow flow can decrease efficiency (Yust et al., 2023). Conversely, in cold brew extraction, grind size has no significant effect on extracted concentrations of ChA or CAF (Yust et al., 2023). Extended cold brew maceration facilitates slow diffusion from intra-particle pores to inter-particle spaces, reaching equilibrium concentrations of ChA and CAF after approximately 400 min, whereas in hot brew, the relationship between CAF concentration and grind size is ambiguous—some studies report higher yields with coarser grinds, others with finer grinds, under identical brewing conditions and contact times (Yust et al., 2023). Severini et al. (2018) demonstrated that grind size exerts a greater influence on extraction kinetics than pressure, coffee dose, or contact time; in espresso (7 g/30 mL, 9 bar), fine grinding (<200 μm) enhances phenolic extraction compared to coarser grinds (Severini et al., 2018).

In light of these findings, our study employed a grind size of approximately 500 μm, static maceration at a 1:20 coffee-to-solvent ratio, and optimized solvent volume. When combined with the dynamic circulation and controlled pressure of Hardtank^®^ technology, this approach yielded extraction efficiencies surpassing all conventional methods.

### 3.4 Antioxidant activity using synthetic DPPH radical and FRAP

**FIGURE 8:**
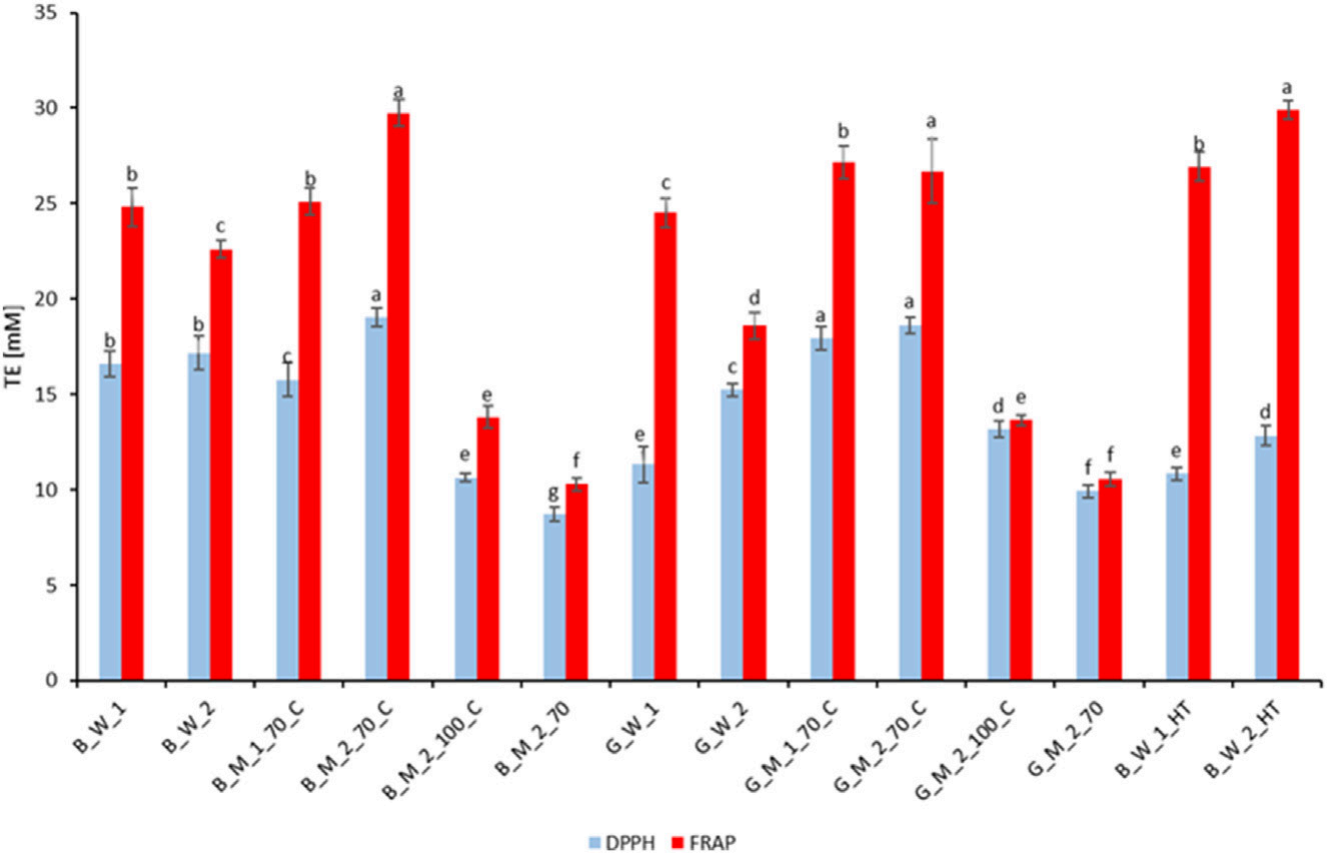
Antioxidant activity of experimental extracts of roasted and green coffee and of the commercial Nitro Cold Brew beverage (HT). Sample codes as in Table 1. Different letters **(a, b, c)** indicate significant differences at p < 0.05.

Figure 8 illustrates the antioxidant activity of water and methanol-water extracts from roasted coffee, green coffee, and the commercial product Nitro Cold Brew (HT).

Our FRAP and DPPH assays confirm that Nitro Cold Brew (HT) extracts rank among the most potent in both reducing capacity and radical-scavenging activity compared to conventional infusions and methanol–water extracts. In the FRAP assay, HT samples B_W_1_HT and B_W_2_HT yielded 26.92 mM and 29.91 mM TE, respectively, matching the highest values obtained by 70% methanol reflux extracts despite requiring only 30–50 min *versus* 6–8 h for the latter. In the DPPH assay, HT beverages (10.83–12.83 mM TE) exhibited slightly lower activity than the B_M_1_70_C reflux extract but substantially outperformed classical water infusions (16.58–17.17 mM TE) and non-refluxed shake extracts (B_M_2_70; 8.72 mM TE).

Interestingly, green coffee extracts displayed a distinct antioxidant profile compared to their roasted counterparts. Traditional water infusions of green beans (G_W_1, G_W_2) produced FRAP values of 24.51 mM and 18.59 mM and DPPH values of 11.32 mM and 15.23 mM, demonstrating respectable reducing power and radical scavenging. The 70% methanol–water reflux extracts of green coffee (G_M_1_70_C: FRAP 27.16 mM, DPPH 17.95 mM; G_M_2_70_C: FRAP 26.69 mM, DPPH 18.63 mM) ranked among the top performers of all samples, underscoring that aqueous methanol with full reflux is highly effective for unroasted beans. By contrast, 100% methanol reflux (G_M_2_100_C: FRAP 13.66 mM, DPPH 13.18 mM) and rapid shake extraction (G_M_2_70: FRAP 10.57 mM, DPPH 9.92 mM) yielded markedly lower values, confirming that water content and extended solvent–particle contact are essential for efficient antioxidant extraction from green coffee.

Overall, green-bean extracts demonstrated antioxidant activities comparable to–and in some cases exceeding–those from roasted-bean extracts. In water infusions, roasted samples slightly outperformed green in FRAP and DPPH, whereas in 70% MeOH reflux, green extracts (FRAP ≈26–27 mM; DPPH ≈18–19 mM) equaled the best roasted extracts. These differences reflect the altered polyphenol and flavonoid profiles caused by roasting–formation of new compounds (e.g., melanoidins) and degradation of heat-sensitive phenolics–which influence extraction kinetics and yield. Under 100% MeOH reflux, FRAP differences were minimal, but green extracts clearly excelled in DPPH, highlighting the higher content of heat-labile flavonoids in unroasted beans. Even in rapid shake extraction, green coffee delivered superior results (FRAP ≈10.6 mM vs. 10.3 mM; DPPH ≈9.9 mM vs. 8.7 mM), emphasizing that raw bean composition critically determines the efficiency of each extraction method.

Antioxidant activity in coffee extracts is closely linked to phenolic compound concentrations (TPC/TFC), as extensively documented in the literature. Cardoso et al. (2020) demonstrated that kombucha brewed from black tea, richer in TPC and TFC, exhibits higher antioxidant activity than green tea infusions, and Zhang et al. (2023) reported a linear relationship between polyphenol content in juices and pomaces and their antioxidant capacity, with pomaces containing up to tenfold higher concentrations of phenolics and flavonoids than the corresponding juices. In our analyses, the elevated TE values in FRAP and DPPH assays for B_M_2_70_C and HT extracts reflect their highest combined TPC + TFC levels, exceeding 395 mg/100 mL (Figure 5). Although many studies confirm a linear correlation between phenolic content and antioxidant activity (Shan et al., 2005; Zheng and Wang, 2001), this relationship is not universally proportional (Kähkönen et al., 1999). Thoo et al. (2010) and Liyana-Pathiran and Shahidi (2005) observed a negative correlation with prolonged maceration times, indicating degradation of certain low-molecular-weight phenolics–such as those in wheat bran–under conditions of high temperature, light, oxygen exposure, or extended extraction, which reduces antioxidant capacity. These findings underscore the advantage of short extraction protocols, such as Hardtank^®^, which minimize bioactive degradation while preserving high antioxidant activity. From a practical perspective, the elevated combined TPC + TFC (>395 mg/100 mL) in HT beverages (B_W_1_HT and B_W_2_HT) and their exceptional FRAP and DPPH values translate into a clear advantage of this technology over traditional coffee antioxidant extraction methods. By employing dynamic circulation, controlled pressure, and optimized solvent parameters, Hardtank^®^ enables the production of functional beverages with high health-promoting potential.

Zhang et al. (2022a) demonstrated that HPP-assisted cold brew (400 MPa, 5 min) accelerates extraction kinetics by approximately 72% and increases FRAP values by around 36% compared to conventional cold brew. Schwarzmann et al. (2022) confirmed that HPP shortens maceration time from 24 h to several–dozens of minutes while maintaining ≥98% of the overall extraction yield. Yust et al. (2023) further showed that, in cold brew (1:20, 6–24 h), FRAP and DPPH activities increase linearly until reaching a plateau at ∼12 h, emphasizing the importance of maceration time for full antioxidant release.

Extraction temperature also influences TPC and TFC levels, which determine antioxidant activity. Rao and Fuller (2018) reported that hot brew (96 °C, 3 min) achieves ABTS activity of ∼18–20 mmol TE/L, whereas cold brew (21 °C–25 °C, 18 h) yields slightly lower values of ∼13–17 mmol TE/L. In our study, shake-extracted samples at room temperature (B_M_2_70 and G_M_2_70) exhibited significantly lower FRAP and DPPH responses compared to their reflux counterparts (B_M_2_70_C and G_M_2_70_C), consistent with their respective TPC and TFC concentrations (Figure 5).

Recent literature emphasizes that, although FRAP and DPPH assays both report results in TE, their differing mechanisms yield non-directly comparable outcomes. Rumpf et al. (2023) found that FRAP and Folin-Ciocalteu–both single-electron transfer (SET)–based assays–correlate most strongly (*R* ^2^ ≈ 0.94), whereas DPPH shows only weak correlation (*R* ^2^ ≈ 0.45), suggesting that DPPH reflects distinct antioxidant properties. Prior et al. (2005) highlighted the need for assay standardization, noting that variations in buffer pH, solvent choice, or incubation time can produce significantly divergent results across ORAC, FRAP, and DPPH assays, even when using the same Trolox calibration curve. Conversely, Śnieżek et al. (2016) observed very high agreement between FRAP and DPPH (r = 0.98) in dried fruit, onion, and garlic matrices, likely owing to both assays’ shared SET mechanism and the high polyphenol content.

Comparative studies further indicate that FRAP–measuring only Fe^3+^ reduction capacity–is more linear, rapid, and less susceptible to color interference than DPPH, which, due to the steric structure of the radical, is less responsive to larger phenolics and reacts more slowly (ABTS >> DPPH in reaction speed; mean times: 12 vs. 30 min). Consequently, FRAP is often used to quantify total reducing potential, while DPPH serves as an indicator of radical-scavenging capacity. Employing both assays provides complementary insights: FRAP for its reproducibility and speed, and DPPH for its information on hydrogen-atom transfer mechanisms.

**FIGURE 9:**
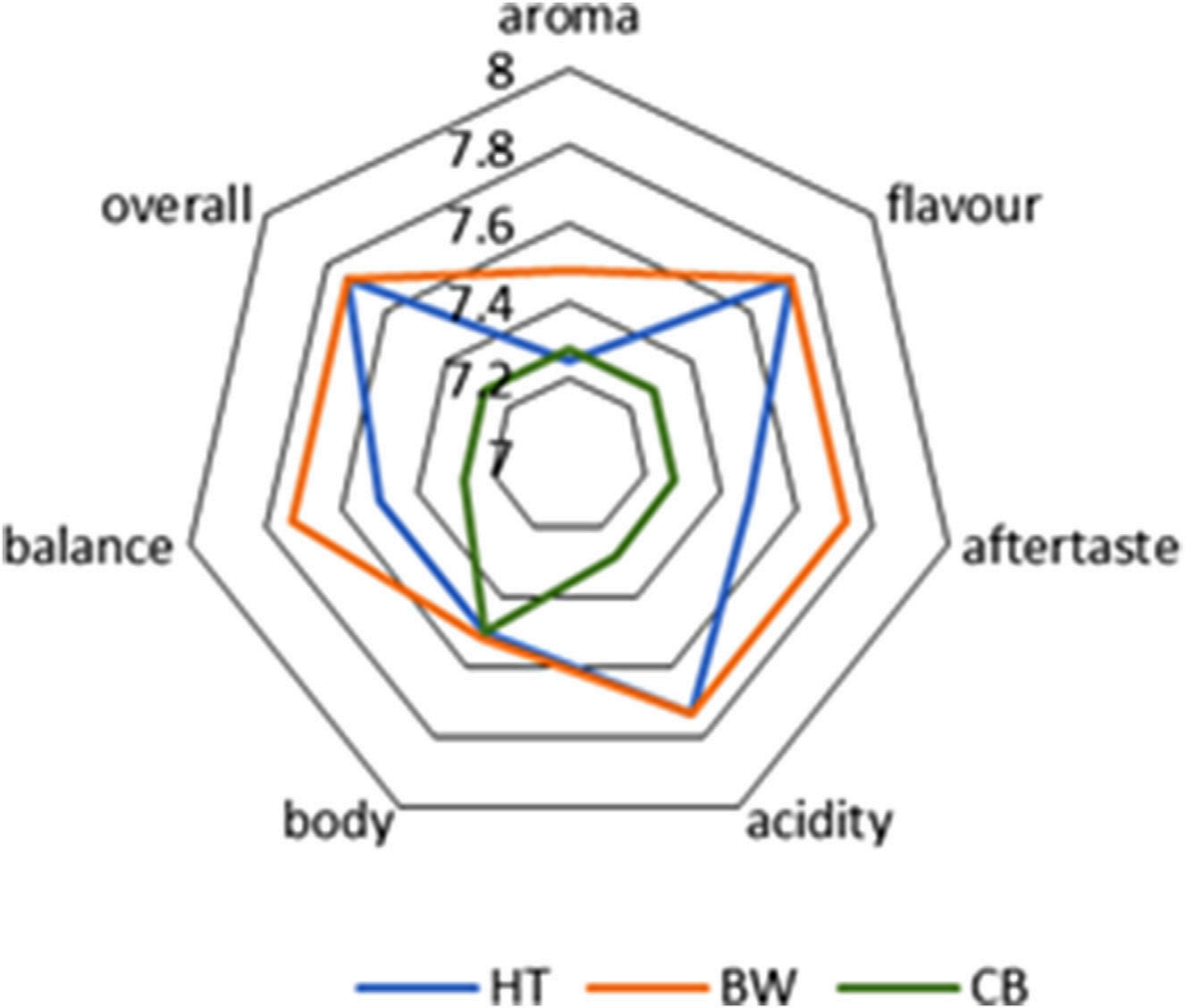
Sensory profiles of coffee samples (Stanek et al., 2021).

In food studies, sensory evaluation plays a critical role, as it is closely linked to both chemical composition and physical parameters. In this work, we draw upon previously conducted sensory tests designed to compare coffee beverages prepared by different methods. Stanek et al. (2021) performed sensory evaluations according to the SCAA protocol on brews prepared from the same coffee variety used here (Yellow Bourbon, pulped natural method, producer Henrique Dias Cambraia, Campo das Vertentes). They compared traditional cold brew (CB), percolated cold brew (Hardtank^®^; HT), and classic hot brew (BW), assessing seven attributes–aroma, flavor, aftertaste, acidity, body, balance, and overall impression–on a 16-point scale (Figure 9).

Stanek et al. (2021) report that coffee beverages produced by the traditional cold brew process exhibited low acidity, a short, sharp aftertaste, and muted, oxidized flavor notes, in contrast to those prepared using the innovative Hardtank^®^ cold extraction method, which displayed pronounced acidity, complex flavor profiles, and a clean, long-lasting aftertaste. Hot-brewed coffees were characterized by intense flavors, a sweet yet sharp finish, and balanced acidity. The sensory analysis demonstrated that Hardtank^®^ extracts received overall scores comparable to hot brews and significantly higher (p < 0.05) than traditional cold brews in aroma, flavor, aftertaste, acidity, and balance. Thus, alongside their favorable chemical composition and high antioxidant potential (TPC, TFC, CAF, ChA, ChAd, FRAP, DPPH), beverages produced by Hardtank^®^ technology also exhibit appealing sensory characteristics.

## 4 Conclusion

This study confirmed that Hardtank^®^ (HT) technology enables solvent-free, ready-to-drink production of Nitro Cold Brew coffee with a phenolic and antioxidant profile that matched or exceeded laboratory reflux extractions, while requiring only 30–50 min. The beverage contained 375.8 ± 2.4 mg TPC + TFC/100 mL, 72.4 ± 1.1 mg CAF/100 mL, 78.2 ± 2.3 mg ChA/100 mL, and exhibited FRAP 27.9 ± 0.5 mM TE and DPPH 11.7 ± 0.6 mM TE. Taking 70% MeOH reflux as the methodological reference (100%), HT achieved 135.3% (TPC + TFC), 119.7% (CAF), 172.3% (ChA), 165.4% (ChA + ChAd) and 154.5% (ChAd), demonstrating superior extraction efficiency without the use of organic solvents. Although 70% MeOH reflux of green beans provided very high FRAP/DPPH values, the HT process applied to roasted beans delivered a commercially viable beverage with a competitive phenolic/antioxidant profile and strong scalability potential.

The main limitations of this study concern the lack of direct bioavailability/bioaccessibility data for HT-extracted compounds and the absence of new sensory evaluations for the final Nitro Cold Brew product. Addressing these limitations should guide future research, which should also explore the optimization of process parameters (pressure, flow rate, particle size) for different bean origins and roast levels, evaluate product stability and the impact of nitrogenation during storage, and confirm sensory quality through updated cupping panels.

Overall, the most important advantage of the Hardtank^®^ system lies in its ability to combine speed, solvent-free operation, and industrial scalability while maintaining a bioactive and antioxidant profile equal to or better than intensive laboratory extractions. This highlights HT as a promising technology for the commercial production of functional coffee beverages.

## Data Availability

The original contributions presented in the study are included in the article/supplementary material, further inquiries can be directed to the corresponding author.
